# Characterization of the Prediabetic State in a Novel Rat Model of Type 2 Diabetes, the ZFDM Rat

**DOI:** 10.1155/2015/261418

**Published:** 2015-04-16

**Authors:** Ghupurjan Gheni, Norihide Yokoi, Masayuki Beppu, Takuro Yamaguchi, Shihomi Hidaka, Ayako Kawabata, Yoshikazu Hoshino, Masayuki Hoshino, Susumu Seino

**Affiliations:** ^1^Division of Molecular and Metabolic Medicine, Department of Physiology and Cell Biology, Kobe University Graduate School of Medicine, Kobe 650-0017, Japan; ^2^Division of Cellular and Molecular Medicine, Department of Physiology and Cell Biology, Kobe University Graduate School of Medicine, Kobe 650-0017, Japan; ^3^Hoshino Laboratory Animals, Inc., Ibaraki 306-0606, Japan

## Abstract

We recently established a novel animal model of obese type 2 diabetes (T2D), the Zucker fatty diabetes mellitus (ZFDM) rat strain harboring the *fatty* mutation (*fa*) in the leptin receptor gene. Here we performed a phenotypic characterization of the strain, focusing mainly on the prediabetic state. At 6–8 weeks of age, *fa/fa* male rats exhibited mild glucose intolerance and severe insulin resistance. Although basal insulin secretion was remarkably high in the isolated pancreatic islets, the responses to both glucose stimulation and the incretin GLP-1 were retained. At 10–12 weeks of age, *fa/fa* male rats exhibited marked glucose intolerance as well as severe insulin resistance similar to that at the earlier age. In the pancreatic islets, the insulin secretory response to glucose stimulation was maintained but the response to the incretin was diminished. In nondiabetic Zucker fatty (ZF) rats, the insulin secretory responses to both glucose stimulation and the incretin in the pancreatic islets were similar to those of ZFDM rats. As islet architecture was destroyed with age in ZFDM rats, a combination of severe insulin resistance, diminished insulin secretory response to incretin, and intrinsic fragility of the islets may cause the development of T2D in this strain.

## 1. Introduction 

Type 2 diabetes (T2D) is the most common form of diabetes, afflicting more than 80% of all people with the disease. T2D is a metabolic disorder characterized by chronic hyperglycemia due to insulin resistance and/or impaired insulin secretion. Despite the increasing incidence and prevalence of T2D, little is known about effective treatment and prevention of the disease and its complications at early stages.

Spontaneous animal models have contributed greatly to the study of T2D. There are several useful rat models such as the Goto-Kakizaki (GK) rat [1], Wistar fatty rat [2], Zucker diabetic fatty (ZDF) rat [3], Otsuka Long-Evans Tokushima fatty (OLETF) rat [4], and Spontaneously Diabetic Torii (SDT) rat [5]. Spontaneous models enable comparison of normoglycemic, prediabetic, and diabetic states in a limited time period and are especially helpful in the study of mechanistic, pathophysiological, and prevention aspects of T2D. Only a few of these models are widely suitable for use in these studies, including the ZDF and the SDT rat.

We recently established a novel rat model of obese T2D, the Zucker fatty diabetes mellitus (ZFDM) rat harboring the* fatty* mutation (*fa*) in the leptin receptor gene.* fa/fa* male rats maintain the normoglycemic state until 7 weeks of age and then develop diabetes as early as 10 weeks of age, reaching 100% incidence at around 20 weeks of age [6]. In contrast to the original Zucker fatty (ZF) rat [7], the Wistar fatty rat, and the ZDF rat,* fa*/*fa* male rats in the ZFDM strain are fertile and possess high reproductive efficiency. ZFDM rats therefore could serve as a useful* model of* T2D.

Here we performed a phenotypic characterization of the ZFDM strain, focusing mainly on the prediabetic state. We also characterized the insulin secretory responses to both glucose stimulation and the incretin GLP-1 in the isolated pancreatic islets. In addition, we compared these characteristics of ZFDM rats with those of original ZF rats.

## 2. Materials and Methods

### 2.1. Animals

Male ZFDM rats (Hos:ZFDM-*Lepr* ^*fa*^,* fa*/*fa* and* fa*/+) were provided by Hoshino Laboratory Animals, Inc. (Ibaraki, Japan). Male ZF rats (Slc:Zucker,* fa*/*fa* and +/+) were purchased from Japan SLC, Inc. (Hamamatsu, Japan). All animals were maintained under specific pathogen free conditions with a 12 h light-dark cycle and were provided with a commercial diet CE-2 (CLEA Japan, Inc., Tokyo, Japan) at the Animal Facility of Kobe Biotechnology Research and Human Resource Development Center of Kobe University. All animal experiments were approved by the Committee on Animal Experimentation of Kobe University and carried out in accordance with the Guidelines for Animal Experimentation at Kobe University.

### 2.2. Oral Glucose Tolerance Test (OGTT)

Glucose (2.0 g/kg body weight) was administered orally to 6 h fasted rats. Blood samples were collected from the tail vein at indicated time points. Blood glucose levels were measured by a portable glucose meter (ANTSENSE III, HORIBA, Ltd., Kyoto, Japan) and plasma insulin levels were measured by insulin ELISA kit (Shibayagi Co., Ltd., Gunma, Japan).

### 2.3. Insulin Tolerance Test (ITT)

Insulin (1.0 IU/kg body weight) (Humulin R, Eli Lilly Japan K.K., Kobe, Japan) was administered subcutaneously to 6 h fasted rats. Blood samples were collected from the tail vein at indicated time points. Blood glucose levels were measured by a portable glucose meter (ANTSENSE III).

### 2.4. Insulin Secretion from Isolated Pancreatic Islets

Pancreatic islets were isolated by the collagenase digestion and Ficoll gradient method [8, 9]. Isolated pancreatic islets were cultured for 3 days in RPMI1640, preincubated for 30 min in H-KRB with 2.8 mM glucose, and then incubated for 30 min in H-KRB with 11.1 mM glucose in the presence or absence of 10 nM GLP-1. Insulin released in the incubation buffer and cellular insulin content in the pancreatic islets were measured by insulin assay kits from CIS Bio international (Gif sur Yvette, France). The amounts of insulin secretion were normalized by the cellular insulin content determined by 0.1% Triton X-100 extraction.

### 2.5. Histological Analysis

Histological analysis of the pancreas was performed using procedures essentially as described previously [10]. Briefly, the pancreas was fixed in 10% neutral buffered formalin. The fixed specimens were embedded in paraffin, sectioned at 4 *μ*m, and stained with hematoxylin and eosin for histopathological examination.

### 2.6. Statistical Analysis

Data are expressed as mean ± SEM. Differences in blood glucose level and plasma insulin level were assessed using Welch's* t*-tests. Differences in insulin secretion from the pancreatic islets were assessed using Tukey-Kramer method. Differences for which the *P* value was <0.05 were regarded as statistically significant.

## 3. Results

### 3.1. Characterization of Glucose Tolerance of the ZFDM Strain

**Figure 1:**
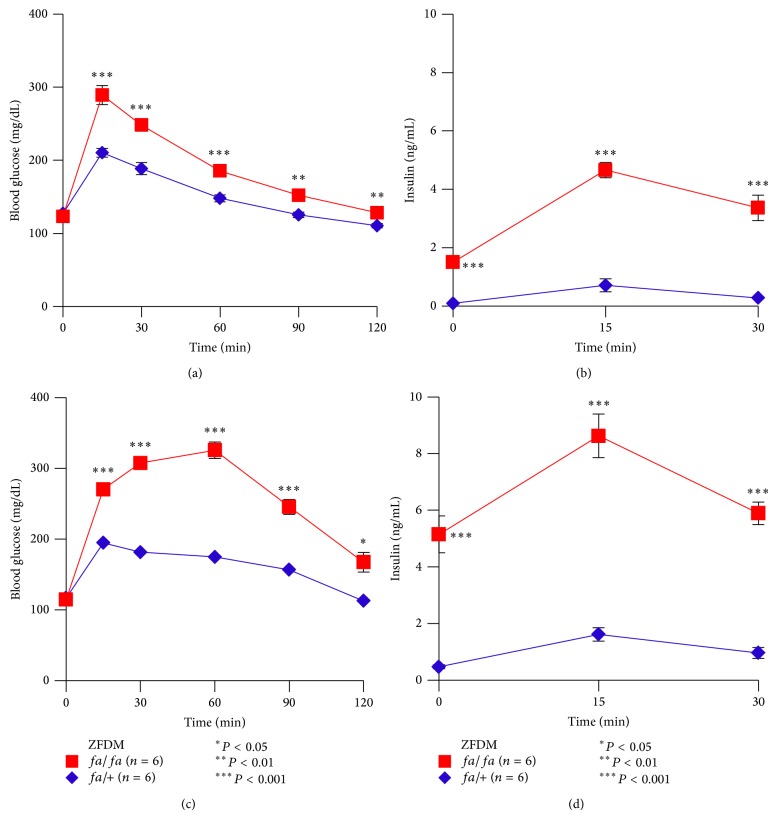
Characterization of glucose tolerance of the ZFDM strain. (a) Blood glucose levels and (b) plasma insulin levels during OGTT at 7 weeks of age. (c) Blood glucose levels and (d) plasma insulin levels during OGTT at 11 weeks of age. The data are expressed as mean ± SEM. Welch's* t*-test was used for comparisons between* fa/fa* (red, *n* = 6) and* fa/+* (blue, *n* = 6) rats.

To characterize glucose tolerance of ZFDM rats at the prediabetic state, we performed OGTT at 6–8 weeks of age and at 10–12 weeks of age. At 7 weeks of age,* fa*/*fa* male rats exhibited mild but significant glucose intolerance after oral glucose loading (Figure 1(a)). At 120 min after glucose loading, blood glucose levels of* fa*/*fa* rats were decreased to the fasting glucose levels. Plasma insulin levels of* fa*/*fa* rats were significantly higher at the fasting state (0 min) than those of* fa/+* rats (Figure 1(b)). The differences in insulin levels were more evident after glucose loading.

At 11 weeks of age,* fa*/*fa* rats exhibited marked glucose intolerance at all time points examined after glucose loading (Figure 1(c)). Blood glucose levels of* fa*/*fa* rats were decreased but not to the fasting glucose levels at 120 min after glucose loading. Plasma insulin levels of* fa*/*fa* rats were markedly higher than those of* fa/+* rats at the fasting state (0 min) and after glucose loading (Figure 1(d)). These results indicate mild glucose intolerance in* fa*/*fa* rats at 7 weeks of age and further deterioration with age.

### 3.2. Characterization of Insulin Sensitivity of the ZFDM Strain

**Figure 2:**
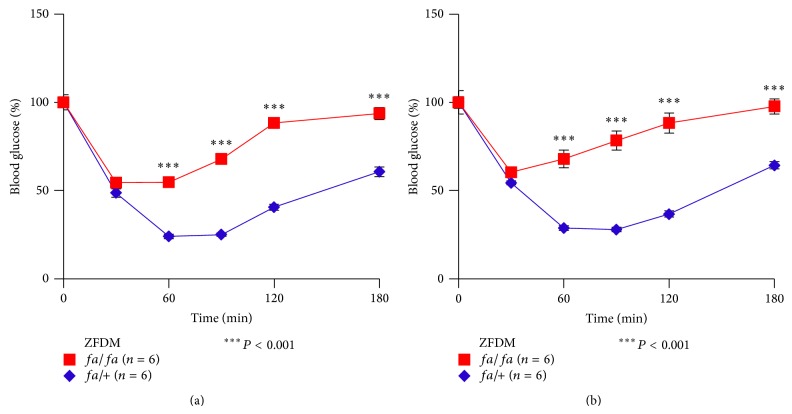
Characterization of insulin sensitivity of the ZFDM strain. Blood glucose levels during ITT at (a) 7 and (b) 11 weeks of age. The data are expressed as mean ± SEM. Welch's* t*-test was used for comparisons between* fa/fa* (red, *n* = 6) and* fa/+* (blue, *n* = 6) rats.

To characterize insulin sensitivity of ZFDM rats at the prediabetic state, we performed ITT at 7 and 11 weeks of age.* fa*/*fa* rats at both ages exhibited severe insulin resistance (Figures 2(a) and 2(b)). There was no apparent difference in the degree of insulin resistance at 7 and 11 weeks of age. These results indicate severe insulin resistance in* fa*/*fa* rats already at 7 weeks, which remains with age.

### 3.3. Comparison of the Glucose Tolerance and Insulin Sensitivity of the ZFDM Strain with the Nondiabetic ZF Strain

**Figure 3:**
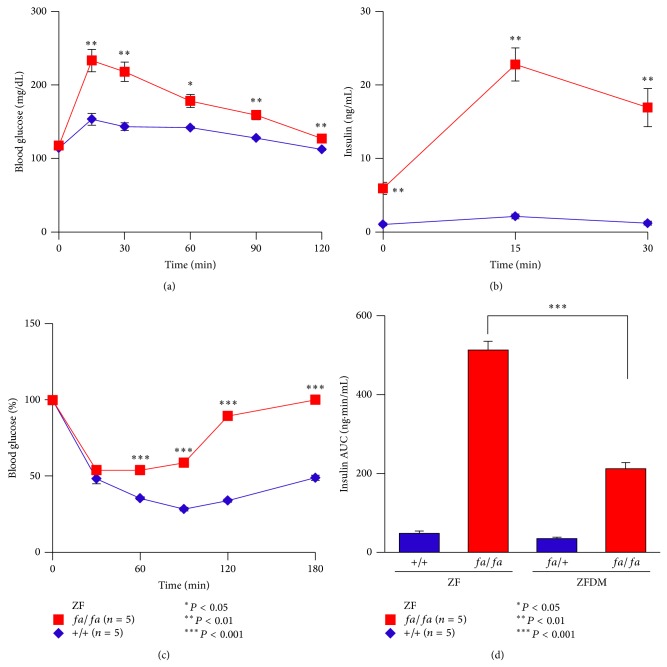
Characterization of glucose tolerance and insulin sensitivity of the ZF strain. (a) Blood glucose levels and (b) plasma insulin levels during OGTT at 12 weeks of age. (c) Blood glucose levels during ITT at 12 weeks of age. (d) AUC data on insulin secretion during OGTT in the ZF and ZFDM strains. The data are expressed as mean ± SEM. Welch's* t*-test was used for comparisons between* fa/fa* (red, *n* = 5) and* +/+* (blue, *n* = 5) rats ((a), (b), and (c)) and between* fa/fa* rats in the both strains (*n* = 5-6) (d).

To compare the features of glucose tolerance and insulin sensitivity of ZFDM rats with those of nondiabetic ZF rats, we performed OGTT and ITT on ZF rats at 12 weeks of age. ZF* fa/fa* rats exhibited mild but significant glucose intolerance after glucose loading (Figure 3(a)). In contrast to ZFDM* fa/fa* rats, blood glucose levels of ZF* fa/fa* rats were decreased to the fasting glucose levels at 120 min after glucose loading. Plasma insulin levels of ZF* fa/fa* rats were significantly higher at the fasting state (0 min) than those of +/+ rats (Figure 3(b)). After glucose loading, the insulin levels of ZF* fa/fa* rats were markedly increased and were significantly higher than those of ZFDM* fa/fa* rats (Figure 3(d)). These findings indicate that* fa*/*fa* rats in the ZF strain can maintain the state of mild glucose intolerance by increasing insulin secretion, while* fa*/*fa* rats in the ZFDM strain cannot, due to defects in insulin secretion.

ZF* fa/fa* rats exhibited severe insulin resistance, as assessed by ITT (Figure 3(c)). There was no apparent difference in the degree of insulin resistance in* fa*/*fa* rats between the ZFDM and ZF strains. These results suggest a similar condition of severe insulin resistance in* fa*/*fa* rats in both strains.

### 3.4. Insulin Secretory Responses to Both Glucose and Incretin Stimulation in Isolated Pancreatic Islets

**Figure 4:**
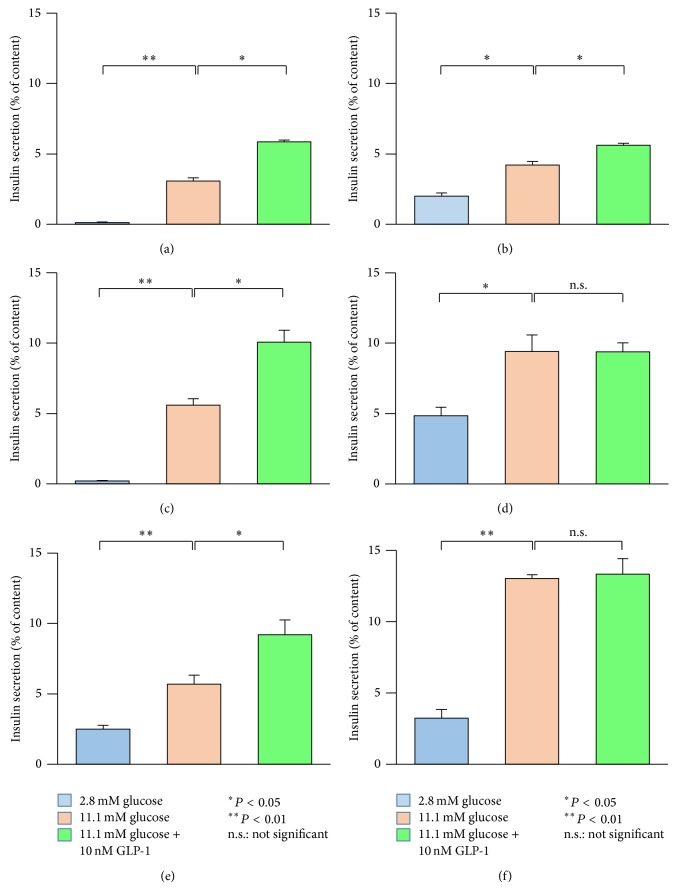
Insulin secretion from the pancreatic islets of ZFDM and ZF strains. Insulin secretory responses to glucose alone (11.1 mM) and glucose plus GLP-1 (10 nM) in (a)* fa/+* (*n* = 6) and (b)* fa/fa* (*n* = 6) rats in ZFDM strain at 7 weeks of age. Insulin secretory responses in (c)* fa/+* (*n* = 6) and (d)* fa/fa* (*n* = 6) rats in ZFDM strain at 11 weeks of age. Insulin secretory responses in (e)* +/+* (*n* = 6) and (f)* fa/fa* (*n* = 6) rats in ZF strain at 11 weeks of age. The data are expressed as mean ± SEM. Differences in insulin secretion from the pancreatic islets were assessed using Tukey-Kramer method.

To clarify the insulin secretory responses to both glucose stimulation and the incretin GLP-1 in pancreatic islets of ZFDM rats at the prediabetic state, we performed batch incubation experiments using isolated pancreatic islets at 7 and 11 weeks of age. Although basal insulin secretion from the pancreatic islets of* fa*/*fa* rats was remarkably higher than that of* fa/+* rats at 7 weeks of age (Figures 4(a) and 4(b)), the insulin secretory responses to both glucose stimulation and incretin were retained in* fa*/*fa* rats. At 11 weeks of age, the insulin secretory response to glucose stimulation was maintained but that to the incretin was extremely diminished in* fa*/*fa* rats (Figures 4(c) and 4(d)). Similar insulin secretory responses were observed in* fa*/*fa* rats in the nondiabetic ZF strain (Figures 4(e) and 4(f)). These findings indicate that defects in insulin secretion from the pancreatic islets are common in* fa*/*fa* rats of both strains.

### 3.5. Histological Characterization of the Pancreas of the ZFDM Strain

**Figure 5:**
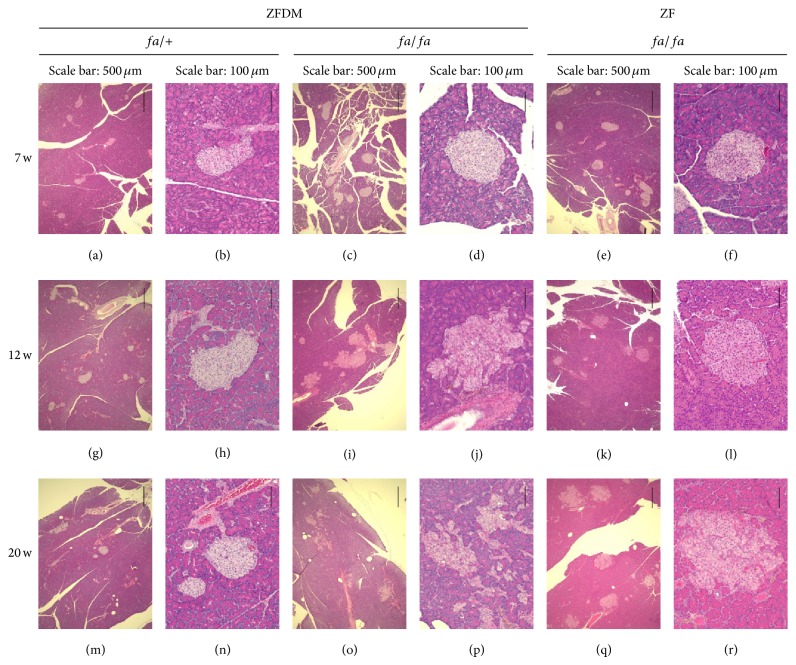
Histological characterization of the pancreas of the ZFDM and ZF strains. Representative pancreas histology of* fa*/+ ((a), (b), (g), (h), (m), and (n)) and* fa*/*fa* ((c), (d), (i), (j), (o), and (p)) male rats in the ZFDM strain and* fa*/*fa* ((e), (f), (k), (l), (q), and (r)) male rats in the ZF strain. Hematoxylin and eosin staining.

To clarify the histological changes in the pancreas of ZFDM rats at the prediabetic state, we performed histological characterization of the pancreas at 7, 12, and 20 weeks of age. There were no obvious pathological changes in endocrine and exocrine pancreas of* fa*/+ rats (Figures 5(a), 5(b), 5(g), 5(h), 5(m), and 5(n)). In* fa*/*fa* rats, enlarged pancreatic islets were observed at 7 weeks of age (Figures 5(c) and 5(d)), and islet architecture was destroyed with age (Figures 5(i), 5(j), 5(o), and 5(p)). In contrast, islet architecture was substantially maintained with age in* fa*/*fa* rats in the ZF strain (Figures 5(e), 5(f), 5(k), 5(l), 5(q), and 5(r)).

## 4. Discussion

In this study, we characterized the prediabetic state of the ZFDM strain. Mild glucose intolerance exists in* fa*/*fa* rats at 7 weeks of age and deteriorates with age. In contrast, severe insulin resistance already exists in* fa*/*fa* rats at 7 weeks of age and remains severe with age. In the pancreatic islets, the insulin secretory response to glucose stimulation is retained but that to the incretin GLP-1 is diminished with age. In contrast,* fa*/*fa* rats in the nondiabetic ZF strain maintain mild glucose intolerance by increasing insulin secretion with age. However, the defect in the insulin secretory response to the incretin is common in* fa*/*fa* rats of both strains. These findings together indicate that, in addition to severe insulin resistance and diminished insulin response to the incretin, other defects are involved in the development of T2D in ZFDM rats.

In the ZFDM strain,* fa*/*fa* rats exhibit enlarged pancreatic islets at 7 weeks of age, in compensation for increased insulin demand due to severe insulin resistance. However, islet architecture is destroyed with age, resulting in relative insulin deficiency. In contrast,* fa*/*fa* rats in the ZF strain maintain enlarged pancreatic islets and architecture with age, which compensates for severe insulin resistance to maintain normoglycemia. These findings suggest that a high degree of fragility of islets is intrinsic to* fa*/*fa* rats in the ZFDM strain. ZFDM* fa/fa* rats fail to compensate for severe insulin resistance, resulting in the development of diabetes.

It has been reported that *β*-cell mass in 5- to 7-week-old prediabetic ZDF* fa*/*fa* rats was similar to that in age-matched ZF* fa*/*fa* rats and greater than that in Zucker lean (+/?) rats [11]. At 12 weeks of age (after diabetes onset), *β*-cell mass in ZDF* fa*/*fa* rats was lower than that that in ZF* fa*/*fa* rats. The failure of *β*-cell expansion was thought to be due to an increased rate of cell death [11]. In another report [12], *β*-cell mass was decreased by 51% from 8 to 12 weeks of age in ZDF* fa*/*fa* rats. The increase in *β*-cell death was well correlated with the increase in plasma glucose levels, suggesting that hyperglycemia in ZDF rats develops concomitantly with increasing net *β*-cell death. Despite the delay in onset of diabetes, a similar change in the *β*-cell mass might occur in ZFDM* fa*/*fa* rats.

We found a diminished insulin response to the incretin GLP-1 not only in ZFDM* fa*/*fa* but in ZF* fa*/*fa* islets at 12 weeks of age, indicating a common defect in the pancreatic islets of both strains. In support of this finding, several pathological features have been reported in ZF* fa*/*fa* islets at 14 weeks of age, such as *β*-cell vacuolation, vascular congestion, haemorrhage, fibrosis, and minimal mononuclear cell infiltration [13]. These pathological changes could affect normal function of the islets, including that of incretin-induced insulin secretion. In addition to relatively large islets accounting for the majority of the islet population, there are relatively small and morphologically normal islets in 11-week-old* fa*/*fa* rats of both strains. Insulin secretory responses to both glucose stimulation and incretin were retained in these small islets (data not shown), which further supports the correlation between pathological changes in the pancreatic islets and the diminished insulin response to incretin.

## 5. Conclusions

In this study, we characterized the prediabetic state of a novel animal model of obese T2D, the ZFDM strain. In addition to severe insulin resistance and diminished insulin response to incretin, intrinsic fragility of islets in ZFDM rats may contribute to the development of T2D in this strain. The ZFDM strain should be useful for studying the mechanisms of incretin-induced insulin secretion and islet fragility in the pathogenesis of T2D.

## References

[1] Goto Y., Kakizaki M., Masaki N. (1975). Spontaneous diabetes produced by selective breeding of normal Wistar rats. *Proceedings of the Japan Academy*.

[2] Ikeda H., Shino A., Matsuo T., Iwatsuka H., Suzuoki Z. (1981). A new genetically obese-hyperglycemic rat (Wistar fatty).. *Diabetes*.

[3] Peterson R. G., Shaw W. N., Neel M. A., Little L. A., Eichberg J. (1990). Zucker diabetic fatty rat as a model for non-insulin-dependent diabetes mellitus. *ILAR Journal*.

[4] Kawano K., Hirashima T., Mori S., Saitoh Y., Kurosumi M., Natori T. (1992). Spontaneous long-term hyperglycemic rat with diabetic complications: Otsuka Long-Evans Tokushima Fatty (OLETF) strain. *Diabetes*.

[5] Shinohara M., Masuyama T., Shoda T. (2000). A new spontaneously diabetic non-obese Torii rat strain with severe ocular complications. *Experimental Diabesity Research*.

[6] Yokoi N., Hoshino M., Hidaka S. (2013). A novel rat model of type 2 diabetes: the Zucker fatty diabetes mellitus ZFDM rat. *Journal of Diabetes Research*.

[7] Zucker L. M., Zucker T. F. (1961). Fatty, a new mutation in the rat. *Journal of Heredity*.

[8] Carter J. D., Dula S. B., Corbin K. L., Wu R., Nunemaker C. S. (2009). A practical guide to rodent islet isolation and assessment. *Biological Procedures Online*.

[9] Yasuda T., Shibasaki T., Minami K. (2010). Rim2*α* determines docking and priming states in insulin granule exocytosis. *Cell Metabolism*.

[10] Yokoi N., Hidaka S., Tanabe S. (2012). Role of major histocompatibility complex class II in the development of autoimmune type 1 diabetes and thyroiditis in rats. *Genes & Immunity*.

[11] Pick A., Clark J., Kubstrup C. (1998). Role of apoptosis in failure of *β*-cell mass compensation for insulin resistance and *β*-cell defects in the male Zucker diabetic fatty rat. *Diabetes*.

[12] Finegood D. T., McArthur M. D., Kojwang D. (2001). *β*-Cell mass dynamics in Zucker diabetic fatty rats. Rosiglitazone prevents the rise in net cell death. *Diabetes*.

[13] Jones H. B., Nugent D., Jenkins R. (2010). Variation in characteristics of islets of Langerhans in insulin-resistant, diabetic and non-diabetic-rat strains. *International Journal of Experimental Pathology*.

